# The role of pericytes on the efficacy of bevacizumab in colorectal cancer

**DOI:** 10.55730/1300-0144.5494

**Published:** 2022-07-09

**Authors:** Merve BESLER, Fatma Sena DOST, Filiz ÇAY ŞENLER, Bilge Ayça KIRMIZI, Berna SAVAS, Hakan AKBULUT

**Affiliations:** 1 Cancer Research Institute, Ankara University, Ankara, Turkey; 2 Department of Medical Oncology, Medical Faculty, Ankara University, Ankara, Turkey; 3 Department of Pathology, Medical Faculty, Ankara University, Ankara, Turkey

**Keywords:** Angiogenesis, bevacizumab, colorectal cancer, pericytes, vascular endothelial growth factor, chondroitin sulfate proteoglycan 4

## Abstract

**Background/aim:**

Pericytes are mesenchymal cells surrounding capillary vessels and are known to play an essential role in tumor angiogenesis. Chondroitin sulfate proteoglycan 4 (CSPG4) is a cell surface proteoglycan and its release from pericytes and vascular smooth muscle cells is very important in tumor angiogenesis. Bevacizumab, which is a monoclonal antibody frequently used in the treatment of metastatic colorectal cancer, binds to the ligand of vascular endothelial growth factor A (VEGFA) and inhibits tumor angiogenesis. However, no reliable biomarker for predicting patients that will show a good response to this therapy has been established yet.

In this study, we aimed to identify the significance of the presence of pericyte and VEGFA and CSPG4 expressions on the efficacy of Bevacizumab.

**Materials and methods:**

Fifty patients with metastatic or recurrent colorectal cancer who had been treated with Bevacizumab combined chemotherapy treatment were included in the study. The expressions of VEGFA and CSPG4 genes and also human β-actin as the reference gene were examined using the quantitative real-time polymerase chain reaction method in the formalin-fixed paraffin-embedded tumor tissues. For determining vascular and pericyte density in tumor tissue, immunohistochemical analysis was performed with CD31, alpha-smooth muscle actin, and CD34 antibodies.

**Results:**

CSPG4 positive group had better objective response rate, as well as longer progression-free and overall survival than CSPG4 negative ones. Progression-free survival was significantly longer in VEGFA low group and CD31 low group. No significant correlation was found between CD34 positivity, SMA positivity, and progression-free and overall survival.

**Conclusion:**

Our results suggested that bevacizumab may be more effective in patients having less vascular density in the tumor tissue. But further studies are needed to support this finding.

## 1. Introduction

Colorectal cancer (CRC) is a significant cause of morbidity and mortality throughout the world [1]. It is the second most common cancer in women and the third most common cancer in men [2]. The median survival time of patients with advanced CRC is around 2 years [3].

In recent years, the addition of chemotherapy to targeted therapies has resulted in some improvement in survival outcomes. Among these drugs, the most commonly used one is bevacizumab, which binds to the ligand of vascular endothelial growth factor A (VEGFA). VEGFA is an essential ligand in angiogenesis. VEGFA enhances capillary permeability and endothelial cell proliferation via VEGFR-2 tyrosine kinase receptors expressed in endothelial cells [4].

Bevacizumab, in combination with chemotherapy, increases the survival rate in patients with metastatic colorectal cancer [5,6]. However, it is useful in only 30%–40% of the patients. Any parameter or marker, which can be used for selecting the patient group that will benefit from bevacizumab therapy, has not been found yet.

Pericytes are mesenchymal cells surrounding capillary vessels and are in close contact with endothelial cells [7]. Pericytes envelop and support endothelial cells and promote vessel growth and maturation. Chondroitin sulfate proteoglycan 4 (CSPG4), which is a member of transmembrane chondroitin sulfate proteoglycans family, is released from pericytes. CSPG4 guides endothelial tip cells to lead to the tubular structure during the angiogenesis in the direction of hypoxic tumor [8]. Therefore, pericytes play an essential role in initiating vascular destabilization and the formation and migration of endothelial end cells in the early stage of tumor angiogenesis. The role of pericytes in the formation of new vessels in tumors has been shown in various studies and the low pericytic area was found to be associated with poor prognosis [9, 10].

With these in the background, we aimed to investigate the effect of the presence of vessel and pericyte density, and VEGFA and CSPG4 expressions in the tumor tissue, on the efficacy of the bevacizumab in metastatic or recurrent colorectal cancer patients.

## 2. Materials and methods

### 2.1. Patients and study design

After the Clinical Research Ethics Committee of Ankara University had approved the study protocol (approval no: 19-963-16), 50 patients with metastatic or recurrent colorectal cancer who had been treated with bevacizumab for at least 2 months in addition to the routine chemotherapy protocol were included. Following the approval, the formalin-fixed paraffin-embedded tumor tissue samples of the patients were used. VEGFA and CSPG4 gene expressions were determined with quantitative real-time PCR, and for determining vascular and pericyte density, immunohistochemical analysis was employed.

### 2.2. Quantitative real-time PCR (qPCR)

Forward and reverse primers and FAM TAMRA stained probes for quantitative real-time PCR (qPCR) analysis of VEGFA and CSPG4 genes were designed with Primer3 web (version 4.0.0) program [11]. Human β-actin (HBA) gene was used as the reference gene for the analysis. Primer BLAST and NCBI BLAST were used to check whether the primers and probes designed are specific to the target gene and control the level of homodimer and heterodimer binding [12,13]. Moreover, IDT OligoAnalyzer 3.1 and PerlPrimer software were used [14,15]. The primer and probe sequences used in qPCR analyses are shown in Table 1.

RNA was extracted from paraffin-embedded blocks of the patients using a commercial kit (RNeasy FFPE Kit; QIAGEN, Hilden, Germany), samples with RNA purity between 2.1 ≥ λ260 and λ280 ≥ 1.8 were used in the study and RNA quality was evaluated through visualization of the 28S:18S ribosomal RNA ratio on a 2% agarose gel. Complementary DNAs (cDNAs) were produced from 1 μg of RNA of each sample using a commercial kit (FIREScript cDNA Synthesis KIT; Solis Biodyne, Estonia) according to the manufacturer’s instructions.

**Figure 1:**
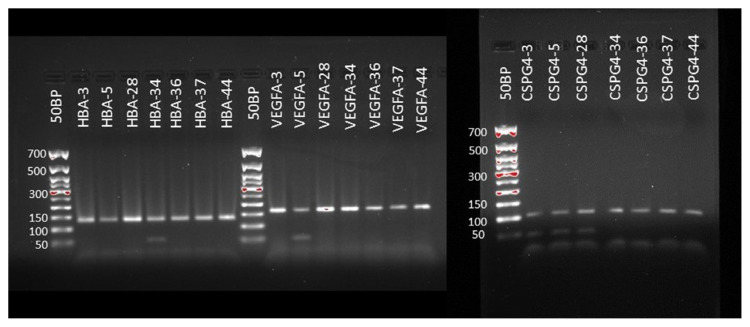
Images of some PCR amplified samples for 2% gel for HBA, VEGF, and CSPG4 genes. HBA product size: 134 bp; VEGFA product size: 161 bp; and CSPG4 product size: 102 bp.

qPCR analysis was performed on CFX96 RealTime System (Biorad C1000 Thermal Cycler, California, USA). Using the qPCR mix, the PCR reaction mixture consisted of 3 μL of cDNA including primers and FAM-TAMRA stained TaqMan probes 0.3 μL (10 pmol/μL), 2 μL of qPCR Mix (Solis Biodyne 5x Hot FirePol Probe Mix Plus (No Rox), Estonia), and 4.1 DNAse-RNAse free water in a final volume of 10 μL per reaction. Cycling conditions were 95 °C for 15 min, followed by 40 cycles at 95 °C for 20 s and 60 °C for 1 min, and then 37 °C for 10 min. The specificity of the qPCR products was confirmed by melting curve analysis and 2% agarose gel electrophoresis (Figure 1). All samples were analyzed in a triple in qPCR assay. The relative quantification of the expression of each gene was calculated by the 2^−Δ(Δct)^ method [16].

### 2.3. Immunohistochemical studies

**Figure 2:**
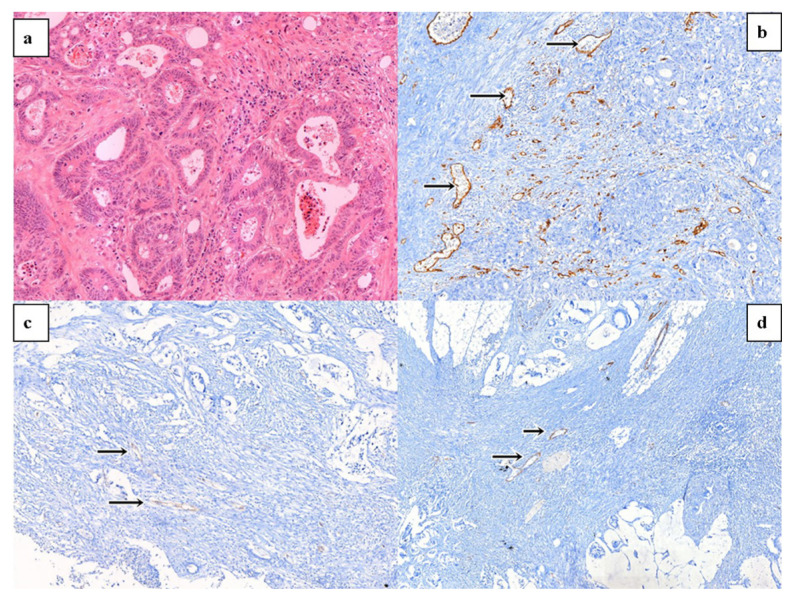
Microscopic pictures of colon adenocarcinoma (a) Tumor development with atypical epithelial cells forming glandular structures, Hematoxylin and eosin stain, 200×, (b) anti-CD31 immunohistochemistry, 200×, (c) anti-CD34 immunohistochemistry, 200×, and (d) anti-alpha SMA immunohistochemistry, 200×. Black arrows indicate the vascular structures.

Hematoxylin & eosin-stained slides (Figure 2a) were reevaluated and the appropriate tumor blocks for the immunohistochemical study were selected. Four-micrometer thick sections were taken and stained with anti-CD31, anti-alpha-SMA, and anti-CD34 antibodies on the Ultraview Universal DAB Detection Kit (Ventana, Cat. No. 05269806001) on a fully-automated immunohistochemical staining device (Ventana, BenchMark ULTRA). Anti-CD31 (Santa Cruz, sc-376764), anti-alpha-SMA (Santa Cruz, sc-53142), and anti-CD34 (Santa Cruz, sc-74499) primary antibodies were used. When evaluating the results of immunohistochemistry, staining of the necrotic areas (if any) and edge regions of the tissues were not taken into consideration considering the possibility of false positivity. The vessels were stained with anti-CD31, anti-CD34, and anti-alpha-SMA immunohistochemically, and the sections were examined at 20× and 100× magnification, and the regions containing the most microvascular structures were determined. The vessels in these regions were counted at 20× magnification and the vascular density was determined (Figures 2b–2d). Analysis was made by using the hotspot Weidner’s method [17].

### 2.4. Statistical analysis

SPSS 10.0 software was used for statistical analysis. Variables were used as mean ± standard deviation and percentage and frequency values. In addition, homogeneity of variances from prerequisites of parametric tests was checked by the Levene test. The test of normality was examined by the Shapiro–Wilk test. For the comparison of two groups, Student’s t-test and Mann–Whitney U test were used.

For CD31, CD34, and anti-alpha SMA, cut-off values were calculated by ROC curve analysis. In the Youden index, the maximum value according to the formula maximum = sensitivity + specificity-1 was taken as cut-off values. Survival analysis was evaluated with the Kaplan–Meier method and the comparison of survival time between the categories of variables was evaluated by the log-rank Mantel–Cox test. In this study, the Cox regression analysis was used to reveal the model of the relationship between independent variables and dependent variables (survival status). The relationships between categorical variables were analyzed with Fisher’s exact test and χ^2^ tests.

## 3. Results

Sex, age, primary tumor localization, disease status, metastasis site, KRAS mutation status, CEA level at diagnosis, Bevacizumab setting, and the chemotherapy schemes administered with bevacizumab are shown in Table 2. Female/male ratio was 0.47. The median age of the patients was 59 years ranging between 34 and 76. Left colon was more frequently seen as the primary tumor site (70%). Sixty-eight percent of the patients had metastasis and 32% had recurrence. Liver was the most frequent metastasis site (80%), followed by lung (28%), lymph node (28%), and peritoneum-omentum-mesentery (18%). Forty-six percent of the tumors showed KRAS mutation, 40% of the tumors was KRAS wild type, and KRAS status was not known in 14% of the tumors. Serum CEA levels were high in 58% of the patients. Bevacizumab was used in the first-line therapy in 78% of the patients, second-line in 10% of the patients, third-line in 8% of the patients, and fourth-line in 4% of the patients. Chemotherapy modalities administered with bevacizumab were as follows; irinotecan-based in 86% of the patients, oxaliplatin-based in 8% of the patients, and fluoropyrimidine in 6% of the patients.

The treatment outcomes of the patients are listed in Table 3. The median number of bevacizumab cycles was 4, ranging between 2 and 18. The objective response rate was 31.1% with a median duration of 5.6 months. The median progression-free survival (PFS) was 8.72 ± 0.8 months (95% confidence interval [CI]: 7.1–10.3), and the median overall survival (OS) was 17.0 ± 2.8 months (95% CI: 11.4 – 12.6).

Quantitative real-time PCR analysis revealed that 20 of the patients (40%) showed VEGFA low expression (VEGFA/HBA<0.5), while 30 of the patients (60%) expressed high VEGFA (VEGFA/HBA>0.5). In addition, 7 of the patients (14%) expressed CSPG4. The objective response rates according to the quantity of VEGFA and the presence of CSPG4 are shown in Table 4. Objective response rates were similar in VEGFA low (39.1%) and VEGFA high (22.7%) (p = 0.235) groups. Besides, there was no statistically significant difference between CSPG4 positive (36.6%) and CSPG4 negative (0%) groups for the objective response rates (p = 0.054).

**Figure 3:**
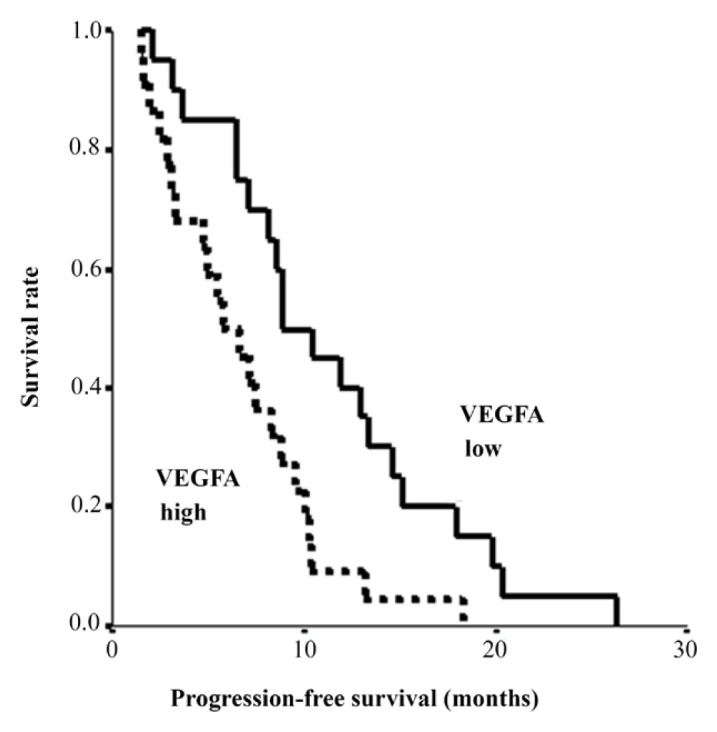
Progression-free survival (PFS) curves according to the quantity of VEGF in primary tumor tissue. PFS was significantly longer in the VEGFA low group (p = 0.0066).

Progression-free and overall survival of the patients was analyzed with the Kaplan–Meier method according to the expression of HBA, VEGFA, and CSPG4 genes. The progression-free survival of the VEGFA high group was less than that of the VEGFA low group (p = 0.0066) (Figure 3). There was no statistically significant difference in overall survival between VEGFA high group and VEGFA low group (p = 0.3036). For CSPG4, the progression-free and overall survival of the CSPG4 positive group seems to be better than the CSPG4 negative group without statistical significance (23.9 ± 18.9 vs 15.4 ± 2.8 months; p = 0.4177, p = 0.5038, respectively).

**Figure 4:**
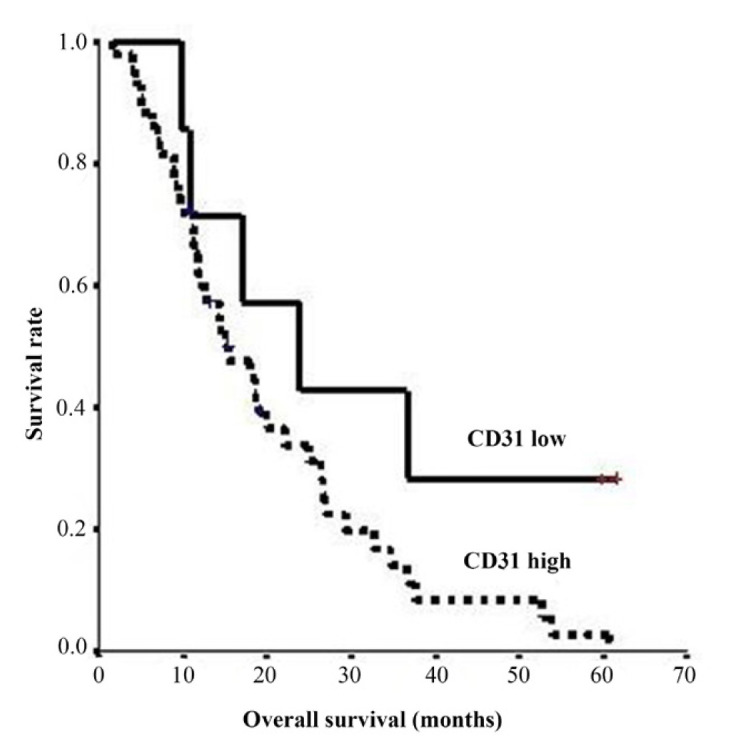
Overall survival (OS) according to the quantity of CD31. The OS for CD31 low group was significantly longer (p = 0.033).

When the results obtained by immunohistochemical staining of CD31, CD34, and alpha-SMA antibodies were evaluated, the median value for vascular density was 48 for CD31, 19.5 for CD34, and 6 for alpha-SMA. According to the Youden index, the cut-off values for vascular density were 39 for CD31, 12.5 for CD34, and 2.5 for alpha-SMA. There was a significant negative correlation between CSPG4 expression and CD31 (r: −0.3428, p: 0.012), whereas a negative correlation was found between CD34 and alpha-SMA expression. There was a weak correlation between VEGF expression and CD34 (r: 0.251, p: 0.096) and SMA (r: 0.273, p: 0.069), but not with CD31. Both progression-free survival (p = 0.042) and overall survival (p = 0.033) were found to be better in those with low CD31 expression (Figure 4). There was no statistically significant difference between CD34 high and CD34 low groups in terms of progression-free and overall survival (p = 0.8508, p = 0.2049, respectively).

## 4. Discussion

Bevacizumab, in combination with chemotherapy, increases the survival rate significantly in patients with metastatic colorectal cancer [5,6]. Although the antiangiogenic drugs improve survival in clinical trials, a large number of patients up to 60% do not benefit from the treatment. More than half of the patients experience treatment toxicity without getting any useful result at all. When treatment costs and vascular complications are considered, it is vital to identify patients who may benefit from antiangiogenic therapies. Although biomarker studies to predict the efficacy of antiangiogenic treatment is continuing, there have been no reliable markers that can be used in the clinic practice yet.

VEGF stimulates the division of endothelial cells in resting tumors and allows them to form new blood vessels [18]. Pericytes and vascular smooth muscle cells have been reported as the sources of VEGF for endothelial cells [19]. Similarly, CSPG4 expression, which is mainly processed by pericytes, is also a very important step in angiogenesis. As a whole, pericyte maturation is an important step in the process of tumor angiogenesis and may provide biomarkers predictive for bevacizumab treatment and targets for drug development [8]. For this reason, it is important to determine the relationship between endothelial cells and pericytes surrounding the tumor vessels and vascular smooth muscle cells.

In the current study, we analyzed the expression of HBA, VEGFA, and CSPG4 genes, and meanwhile evaluated the vascular and pericyte density with immunohistochemical analysis of 50 colorectal patients’ tumor tissue. Although the number of patients in our study was small and the treatment groups were heterogeneous, there were no significant differences in terms of the treatment in metastatic colon cancer.

No statistically significant difference was found for VEGFA expression for objective response rate and overall survival time, but progression-free survival was significantly longer in the low VEGF expression group, pointing that the quantity of VEGF expression could be used to assume prognosis.

A few clinical studies in the literature indicated that low pericyte density was more associated with poor prognosis [10] and decreased pericytes in the tumor tissue have been reported to support increased rate of metastasis [20]. In a subsequent study, CD31 and alpha-SMA antibodies were stained in metastatic CRC patients with the immunohistochemical method. They have suggested that perivascular cells could be a predictive marker in the treatment of bevacizumab. [21].

In our study we evaluated the CSPG4 expressions for having an idea of the pericytic density in the tumor tissue. The objective response rate was better in CSPG4 positive group, as well as progression-free, and overall survival times without statistical significance, remarking the need of larger series to clarify or exclude these results.

There was no correlation between CD34 and SMA positivity and progression-free and overall survivals. Both progression-free and overall survival were found to be better in the group with low CD31, indicating that immunohistochemical evaluation of CD31 may be useful in determining the efficacy of treatment in patients receiving bevacizumab.In conclusion, our results suggested that bevacizumab may be more effective in patients with less vascular density. However, further studies are needed to evaluate the emerging role of pericytes on the efficacy of bevacizumab.

## Figures and Tables

**Table 1:** Designed primer and probe sequences.

Gene	Forward primer	Reverse primer	Probe
**HBA**	5′-CCTGGAGAAGAGCTACGAG-3′	5′-AAGGTAGTTTCGTGGATGCC-3′	5′-CCAGCCTTCCTTCCTGGGCA-3′
**VEGFA**	5′-TACTGCCATCCAATCGAGAC-3′	5′-GAGGAGTCCAACATCACCAT-3′	5′- ATGCGGGGGCTGCTGCAATGA-3′
**CSPG4**	5′-TGACCCTGACTATGTTGGCC-3′	5′-GCAGGTCTATGTCGGTCAGA-3′	5′-CCGCGGCTTCCTTCTTCGGTGA-3′

**Table 2:** Patient characteristics.

Parameter	

**Sex**	n (%)
Female	16 (32%)
Male	34 (68%)

**Age**	
Median (range)	59 (34–76)
<50 n (%)	13 (26%)
>50 n (%)	37 (74%)

**Tumor localization**	n (%)
Right colon	15 (30%)
Left colon	35 (70%)

**Disease status**	n (%)
Metastatic	34 (68%)
Recurrence	16 (32%)

**Metastasis site**	n (%)
Liver	40 (80%)
Lung	14 (28%)
Lymph node	14 (28%)
Peritoneum-omentum-mesentery	9 (18%)

**KRAS mutation status**	n (%)
Mutant	23 (46%)
Wild type	20 (40%)
Unknown	7 (14%)

**CEA level at diagnosis**	n (%)
High	29 (58%)
Normal	21 (42%)

**Bevacizumab setting**	n (%)
First line	39 (78%)
Second line	5 (10%)
Third line	4 (8%)
Fourth line	2 (4%)
	

**Chemotherapy**	n (%)
Irinotecan/5-FU-based	43 (86%)
Oxaliplatin/5-FU-based	4 (8%)
Fluoropyrimidine	3 (6%)

**Table 3:** Treatment outcomes.

Parameter	

**Bevacizumab cycles**	
Median (range)	4 (2–18)

**Objective response rate**	31.1%

**Response duration, months**	
Median ± SEM	5.6 ± 0.4
95% CI	4.7–6.5

**Progression-free survival, months**	
Median ± SEM	8.72 ± 0.8
%95 CI	7.1 – 10.3

**Overall survival, months**	
Median ± SEM	17.0 ± 2.8
95% CI	11.4–12.6

SEM: Standard error of mean, CI: Confidence interval

**Table 4:** Objective Response Rate for VEGFA and CSPG4.

	Objective response rate	p-value

VEGFA low (VEGFA/HBA < 0.5)	39.1%	0.235
VEGFA high (VEGFA/HBA > 0.5)	22.7%	

CSPG4-positive	36.6%	0.054
CSPG4-negative	0%	
